# Analysis of Fracture Incidence in 135 Patients With Pregnancy and Lactation Osteoporosis (PLO)

**DOI:** 10.7759/cureus.19011

**Published:** 2021-10-24

**Authors:** Brittany Miles, Megna Panchbhavi, James D Mackey

**Affiliations:** 1 Medicine, University of Texas Medical Branch, Galveston, USA; 2 Orthopaedic Surgery and Rehabilitation, University of Texas Medical Branch, Galveston, USA; 3 Medical Oncology, Baylor University Medical Center, Dallas, USA

**Keywords:** parathyroid hormone-related peptide (pthrp), pregnancy and lactation osteoporosis (plo), postpartum osteoporosis, premenopausal osteoporosis, fracture incidence

## Abstract

Pregnancy and lactation-related osteoporosis (PLO) is the development of osteoporosis in a premenopausal woman, usually in the third trimester of pregnancy or puerperium. The hormonal changes that allow for the maternal-fetal calcium gradient may be the underlying cause for bone loss, but it is not currently known why some women are affected so severely. Because osteoporosis does not cause symptoms until the condition is advanced, diagnosis is usually made upon the development of an osteoporotic fracture or incidentally when imaging is performed for other reasons. Spontaneous recovery is common once lactation is discontinued, as the underlying hormonal factors that caused the osteoporosis revert to the pre-pregnancy state.

We used the research database TriNetX (TriNetX, LLC, Cambridge, MA) to perform a query selecting women between the ages of 10 and 50 years old who experienced an osteoporotic fracture within 12 months of pregnancy. We analyzed the cohort of patients to determine the incidence of fractures at different skeletal locations and evaluated the medications that were utilized in the patients who received treatment.

## Introduction

Pregnancy and lactation-related osteoporosis (PLO) is a rare condition which has the potential to cause osteoporotic fractures in premenopausal women in the third trimester of pregnancy or in the early postpartum period. Vertebral bodies are known to be the most common site of fracture but the incidence of other fracture locations is not well understood [1-3]. It is believed that bone loss from this condition may occur from increased levels of parathyroid hormone-related peptide (PTHrP), which generates the maternal-fetal calcium gradient to allow calcium transfer to the fetus [4]. Once lactation is discontinued, the underlying factors that cause osteoporosis are no longer present and spontaneous recovery is expected. Treatment is generally offered only to the most severe cases [5]. Risk factors for the development of PLO are not yet known, although one study has shown that women who experience it may have osteoblast dysfunction more than a year postpartum and that this dysfunction could cause them to respond less well to medical intervention [6].

## Materials and methods

We used TriNetX (TriNetX, LLC, Cambridge, MA), a global federated health research network providing access to electronic medical records (diagnoses, procedures, medications, laboratory values, genomic information) from approximately 64 million patients in 45 large healthcare organizations. The TriNetX platform only uses aggregated counts and statistical summaries of de-identified information. No protected health information (PHI) or personal data is made available to the users of the platform. We performed a query designed to select women between the ages of 10 and 50 years old who experienced an osteoporotic fracture within 12 months of pregnancy. Pregnancy was defined by the ICD-10 codes O00-O9A for pregnancy, childbirth, and the puerperium, and the code Z33 for the pregnant state [7]. The ICD-10 code M80 was used to identify patients having “osteoporosis with current pathologic fracture” (osteoporosis is defined as a bone density that is greater than 2.5 standard deviations below the mean). This resulted in a cohort of 135 patients for whom we were able to explore the diagnosis codes and fracture incidence. A second exploration was performed to evaluate whether patients received treatment for PLO, and what type of therapy was utilized.

Patient case

A recently postpartum 30-year-old woman presented to the office with PLO. She developed back pain in the third trimester of the pregnancy, and postpartum MRI studies revealed advanced osteoporosis with mild compression fractures at several levels of thoracic vertebrae and throughout the lumbar spine. She was managed with a back brace and analgesics. Bone density testing revealed a T-score and Z-score of -2.8 (osteoporotic range) in the lumbar spine, but hip density was normal, and the bilateral femurs were osteopenic. In bone densitometry, the Z-score is a comparison of bone density to age-matched controls, while the T-score is a comparison to the average bone density of a healthy person of the same gender at age 30. Since the patient was 30 years old, both values were the same. She was advised not to breastfeed and opted for calcium and vitamin D supplementation instead of bisphosphonate or denosumab therapy for the treatment of osteoporosis.

## Results

Our analysis revealed that lumbosacral fractures were the most common (44%), followed by fractures of the thoracic spine and ribs (41%) and the cervical spine (27%). Femur, foot/toe, and upper arm fractures were equivalent in incidence at 14% each (Figure 1). Lower leg/ankle and wrist/hand fractures were the least common at 10% and 7%, respectively.

**Table 1:** Fracture Incidence in Pregnancy and Lactation-associated Osteoporosis (PLO) Patients

Location	Number of Patients	Percentage
Fracture of the lumbar spine and pelvis	59	44%
Lumbar vertebra	45	33%
Sacrum	24	18%
Coccyx	23	17%
Fracture of the rib(s), sternum, and thorax	55	41%
Fracture of the thoracic vertebra	46	34%
Fracture of one rib	15	11%
Multiple fractures of the ribs	14	10%
Fracture of the cervical vertebra and other parts of the neck	36	27%
Fracture of the femur	19	14%
Fracture of the foot and toe, except ankle	19	14%
Fracture of the shoulder and upper arm	19	14%
Fracture of the lower leg, including ankle	13	10%
Fracture at the wrist and hand level	10	7%

We then explored the cohort for medications used to treat osteoporosis. We found 10 therapies used with equal frequency (Figure 2).

**Table 2:** Osteoporosis Treatments Used in This Cohort

Medication	Number of Patients	Percentage
Pamidronate	10	7%
Ibandronate	10	7%
Salmon calcitonin	10	7%
Cinacalcet	10	7%
Etidronate	10	7%
Alendronate	10	7%
Risedronate	10	7%
Zoledronic acid	10	7%
Denosumab	10	7%
Teriparatide	10	7%

## Discussion

PLO is a rare condition, and as such, it has been difficult to determine fracture patterns and treatment trends because formal studies would likely suffer from poor accrual. Much of the current literature on PLO is, therefore, limited to case reports or a general description of the disease state, without conclusive recommendations regarding when or how to treat the patients that develop it [1-4, 6, 8-11]. The use of TriNetX has made it easier to aggregate data and overcome the obstacles of rarity and geography for studying rare conditions, such as PLO. However, our study is not without limitations. We were unable to determine whether the trauma was a contributing factor to bone injury and did not exclude other potential confounding causes for osteoporosis, such as hyperparathyroidism, hyperthyroidism, or medications. For patients who received treatment for PLO, we were also not able to determine the duration of treatment. However, all patients were osteoporotic, premenopausal, and either pregnant or within 12 months of delivery. Our data illustrating the predominance of axial fractures is consistent with results from other authors [8-14]. However, this is the first study to evaluate the incidence of femur, foot, arm, and hand fractures with this condition.

The optimal medication and treatment duration for PLO is also currently unknown. After delivery and cessation of lactation, many women with PLO tend to have substantial improvement in the first 12 to 18 months without intervention. Those with more severe osteoporosis who may benefit from treatment may, therefore, only need it for a brief time. One study of PLO patients showed that those who received recombinant human parathyroid hormone (teriparatide) experienced approximately double the improvement of the bone mineral density of the lumbosacral spine compared to those who were not treated [15]. The downside to this approach is that teriparatide is a daily subcutaneous injection which may have convenience, cost, comfort, and compliance considerations for many patients.

A Swedish study of senile osteoporosis patients evaluated the benefit of different oral bisphosphonate treatment durations and found that fracture risk in the first six months after discontinuance was 2.26% in women who had less than one month of treatment versus 1.16% in women who were treated for greater than one year [16]. However, it has also been shown that a single dose of intravenous zoledronic acid may suppress biochemical markers of bone resorption for up to 12 months [17]. In PLO patients, there is concern that, because bisphosphonates are incorporated into the bone matrix, there is a theoretical risk for adverse consequences in future pregnancies [18]. Adverse outcomes have been seen in animal studies, but human studies have been inconclusive because of the small sample size and confounding variables [19]. Bisphosphonate that has deposited into bone will be released based on the underlying rate of bone turnover, resulting in a half-life that can vary from between one and 10 years [20]. Calcitonin, denosumab, and teriparatide are believed to be reasonable alternatives for patients who could benefit from treatment while avoiding theoretical concerns about long-term consequences from bisphosphonate bone deposition. Denosumab, a monoclonal antibody directed against receptor activator of nuclear factor kappa-B ligand (RANKL), could be considered one of the better options for PLO patients as it is a reversible therapy that does not remain in the body long-term and may require only one or two administrations over a 12-month period in these patients.

## Conclusions

Our research confirms that the most common sites of fracture in PLO are axial, but a significant percentage of patients sustain fractures to the extremities, including the hands and feet. Clinicians should be aware that any fracture which occurs within 12 months of delivery may potentially be associated with PLO, and evaluation for the presence of osteoporosis is warranted. Because many cases are self-limited, treatment of PLO is controversial regarding when it should be performed and what medication should be used. There is currently no standard of care but the choice of the agent from the available options may take into consideration both the magnitude of the osteoporosis and the mother’s desire, or lack thereof, for additional children. The safest treatment options at this time include calcitonin, teriparatide, and denosumab because they do not incorporate into bone and, therefore, have less theoretical long-term risk. We believe that denosumab may have the most desirable combination of efficacy, convenience, and lack of long-term risk for those patients who may benefit from treatment.
